# Testicular Biopsy in Males With Infertility: A Longitudinal Study

**Published:** 2017-04-01

**Authors:** Sepideh Siadati, Hamid Shafi, Hossein Ghorbani

**Affiliations:** 1 *Pathology Department,* * Babol University of Medical Sciences, Babol, Iran*; 2 *Urology Department,* * Babol University of Medical Sciences, Babol, Iran*

## Abstract

**Background & objective:**

Regarding the importance of histologic examination of testicular biopsy for clinical planning of infertility, the current study was conducted to compare 2 separate histologic examination of testicular biopsy. Also, some cases with known fertility outcome were followed and their histological patterns were also compared with those of the outcome.

**Methods:**

The current study was conducted on testicular biopsies of 924 males evaluated for infertility from 1990 to 2013, retrieved from the archive of pathology department of Shahid Beheshti Hospital, Babol, Northern Iran. All slides were reviewed by a pathologist unaware of the original results. Data including age, histological pattern of spermatogenesis (pure and mixed), smoking, and the history of ejaculatory duct obstruction were retrieved from the pathology archive. In some cases, the outcome was also compared with that of the histological pattern. All analyses were executed using SPSS version 22 statistical software. To analyze the data, *t* test, Chi-square test, one-way ANOVA, and the least significant difference (LSD) test were used.

**Results and Conclusion:**

Out of the 924 testicular biopsies, 34 (3.7%) cases had different reports from original reading. LSD analysis indicated Sertoli cell only syndrome (SCO) as the most common histological pattern. There was a significant difference between the mean age of cases with SCO and that of the ones with hypospermatogenesis (HYPO) (P =0.03). Obstruction was higher in pure pattern (P=0.04). The pregnancy rate was higher in the wives of males with obstructive infertility than the ones with non-obstructive infertility. SCO was the most common histological pattern of testicular biopsy during 23 years. Pure patterns were more than mixed patterns, and the mean age was lower in mixed patterns. Also, pure patterns were the most common findings in the cases with obstructive infertility.

## Introduction

In infertile couples, 50% of the cases are due to male, out of which, 10% to 20% have azoospermia (1). In males with azoospermia referring to reproductive centers, testicular biopsy and histological examination are the classic mean to distinguish obstructive from non-obstructive cases and to evaluate the histological pattern of seminiferous tubules with respect to different spermatogenesis stages.

Several studies already demonstrated that sperm retrieval for in vitro fertilization (IVF) and intracytoplasmic sperm injection (ICSI) can be predicted by the histological pattern of testis biopsy (2). Successful sperm retrieval is reported in 5% to 60% of the cases (3). Although this can be attributed to differences between procedures among infertility centers, some reports demonstrated interobserver variability in interpretation of testicular biopsies (1, 2). This interpretation is subjective; meanwhile, no uniform standard protocol was used by pathologist. Several algorithms for testicular biopsy reading are used by pathologists worldwide (4). Regarding the importance of histological examination of testicular biopsy for clinical planning, the current study was conducted to compare 2 separate histological examinations of testicular biopsy. Also, some cases with known fertility outcome were followed, and their histological patterns were compared with those of the outcome.

## Material and Methods

The current study was conducted on testicular biopsies of 924 males evaluated for infertility from 1990 to 2013, retrieved from the archive of pathology department of Shahid Beheshti Hospital, Babol, Northern Iran. All slides were reviewed by a pathologist unaware of the original results. Presence of fixation artifact and inadequate specimen (<25 seminiferous tubule cross section) were considered as exclusion criteria. Each slide was evaluated and its histological pattern was described. In the present study, testicular histological patterns were categorized as: normal spermatogenesis (NS), Sertoli cell only syndrome (SCO), hpospermatogenesis (HYPO), complete maturation arrest (CMA), incomplete maturation arrest (IMA), end stage (ES), Klinefelter Syndrome (KFS), and immature testis (IT). The term mixed pattern (MP) was used if 2 or more patterns were observed.

**Statistical Approach**

Pathological and clinical characteristics of the patients were reported. All analyses were executed using SPSS version 22 statistical software. Mean values and related standard deviations for continuous variables, and frequency (%) for categorical data were reported. To analyze the data, *t* test, Chi-square test, one-way ANOVA, and LSD test were used. P-value <0.05 was considered as significant level.

## Results and Discussion

A total of 924 testicular biopsies were submitted to Shahid Beheshti Hospital, Babol, Northern Iran, from 1990 to 2013. All slides were reviewed by a pathologist. The results of 890 (96.3%) cases were similar to the previous report, and 34 (3.7%) cases had different reports from the original reading. The mean age of patients was 29.27.95 years. Basic characteristics of specimens are shown in Table 1.

**Table 1:** Basic Characteristics of Specimens

Variables	N (%)
Side	
Right Left Bilateral	660(71.4) 236(25.5) 28(3.1)
Obstruction	
Yes No	165(17.9) 759(82.1)

**Fig1:**
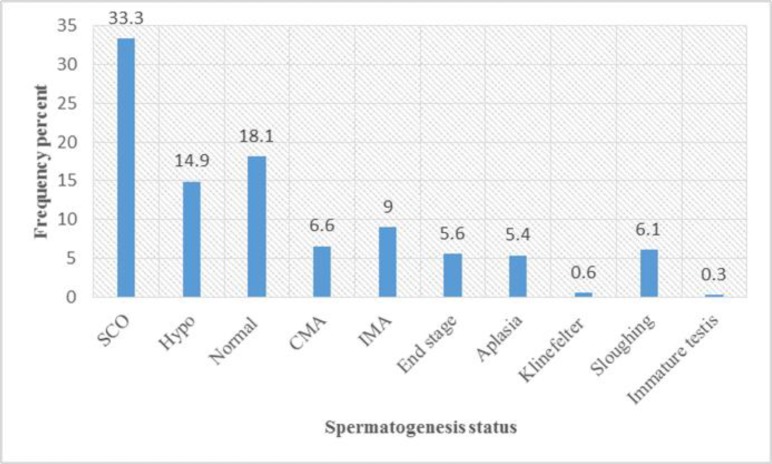
Distribution of Spermatogenesis

The spermatogenesis status is shown in Figure 1. SCO (308 cases) was the most common pattern. 

The mean age of patients with different histological patterns is show in Table 2. 

SCO, 308 (33.3%) cases, constituted the majority of the pure pattern, based on histological information. Sloughing+ other with 56 (43.3%+15.2%) cases was the most common mixed pattern.

Patients with mixed pattern were older than patients with pure histology; however, this difference was statistically insignificant (P=0.61). No significant difference was observed between age and spermatogenesis status in pure pattern (P =0.09). LSD analysis indicated significant difference between the means age of cases with SCO and that of the ones with HYPO (P =0.03). Also, there was a significant difference between the mean age of the cases with SCO and that of the ones with NS (p=0.006). There was no significant difference between the age and spermatogenesis in mixed pattern (P=0.07). LSD indicated a significant difference between the mean age of the cases with HYPO and that of the ones with sloughing (P =0.02). The mean age of the cases with sloughing was higher than that of the ones with HYPO (P=0.02).

**Table 2:** Age Distribution of Different Testicular Histological Patterns

Histology	N (%)	Mean ± SD(years)
Pure patterns	795(86)	28.96±7.88
Normal spermatogenesis	161(20.3)	30.19±9.26
Sertoli cell only syndrome	308(38.7)	28.07±7.70
Hypospermatogenesis	133(16.7)	29.77±8.21
Complete maturation arrest	61(7.7)	28.20±6.09
Incomplete maturation arrest	71(8.9)	28.35±5.31
End stage	52(6.5)	30.17±8.08
Klinefelter syndrome	6(0.8)	27.17±5.56
Immature testis	3(0.4)	32.00±1.00
Mixed patterns	129(14)	29.36±8.42
HYPO + other	29(22.5(	26.83±4.87
End stage + other	6(4.7)	33.50±2.02
IMA + other	17(13.2)	26.71±5.22
Aplasia + other	11(8.5)	26.91±4.82
CMA + other	10(7.8)	30.90±5.99
Sloughing + other	56(43.4)	31.23±8.08

HYPO, hypospermatogenesis; IMA, maturation arrest; CMA, complete maturation arrest

There was no significant differences between the side of biopsy and histological pattern (P =0.21). Obstruction was higher in pure pattern, compared with the mixed pattern, and the difference was statistically significant (P =0.04). Comparing the smoking status, it was concluded that individuals with mixed pattern smoked more 

(P =0.03). Out of 142 available information about the causes of biopsy, retrograde ejaculation and vas deferens obstruction were the most frequent ones. There was no significant association between obstruction and histological pattern (P=0.25) (Table 3). 

**Table 3:** Clinical and Behavioral Characteristics of the Study Cases

Variables	Pure N (%)	Mixed N (%)	P-value
Side			0.21
** Right**	560(84.8)	100(15.2)	
** Left**	209(88.6)	27(11.4)	
** Bilateral**	26(92.9)	2(7.1)	
Obstruction (yes)	149(90.3)	16(9.7)	0.04
Smoking^1^ )yes)	140(80.9)	33(19.1)	0.03
Cause ^2^			0.25
** Epididymal obstruction due to infection**	12(100)	-	
** Vas deferens obstruction**	50(94.3)	3(5.7)	
** Congenital bilateral absence of vas deferens**	20(87)	3(13)	
** Retrograde ejaculation**	46(85.2)	8(14.8)	

1 available information on 524 cases

2 available information on 142 cases

Infertility is one of the important problems worldwide. According to World Health Organization (WHO), more than 70 million couples in the world, and 1.5 million couples in Iran have this problem (5). 

In the current study, the mean age of patients was under 30 years; about 71.4% of biopsies were from right testis. Thirty-seven percent of patients had vas deferens obstruction, out of which 86% had pure histology. SCO with the prevalence of 33.3% was the most common diagnosis. 

Mozaffari et al., reported SCO as the most common pattern that was similar to the results of the current study. The current study results were in agreement with the results of the studies in other countries(6). In the study by Khoei et al., the most common histological pattern was SCO (7). In the survey by Moein and Hussein, spermatic arrest was the most common histological feature (8, 9). In the studies by Kim and Aboutaleb, HYPO was the most frequent pattern (10, 11). These differences could be due to genetic and environmental factors. 

In the current study, the mean age of patients was 29 years. However, Khoei et al., reported the mean age of 27 years, which was lower than that of the current study (7). Infertility has affected 5% to 7% of male population and is increasing (12). In the study by Yousefi et al., the mean age of males with infertility was 35 years, and Ahmadi et al., reported 38 years (13, 14). It seems that this difference was due to different populations. In addition, some studies were performed in rural areas with lower age of marriage. In pure patterns, patients with IT had the oldest mean age; NS and ES with the mean age of 30 years were in the second order, and KFS had the lowest mean age. Considering that the oldest mean age was in mixed pattern, the mean age of ES + other was high. Fertility in males was related to the number and quality of the spermatozoids; therefore, aging could affect semen volume and influence males fertility (15). Different studies showed that aging could reduce the fertility in both males and females. As various factors contribute to these findings, a definite conclusion cannot be made regarding the reasons for different mean ages of histological patterns. It seems that a meticulous investigation is needed, however, the mean age should be discussed further. 

In the current study, mostly the right testis of the patients was biopsied and accordingly, all histological patterns were observed in the right testis. Therefore, no bilateral cases of KFS and IT were reported. In agreement with the results of the current study, Mozaffari et al. reported 28.8% bilateral biopsy. There was no association between the side of biopsied testis and the spermatogenesis; thus, different histological patterns were side independent. Also, 94.3% of the patients with vas deferens obstruction had pure histological pattern. Testis abnormalities are observed in 1% of male population; however, it is 10% in infertile males. 

There are a lot of causes that lead to male infertility, although some are recognized (16). Azoospermia might be due to obstructive or non-obstructive causes detected by testicular biopsy (17). In the present study, 17% of patients had obstructive azoospermia and 81.2% and 4.2% of them showed NS and HYPO patterns, respectively. Najafipour et al., showed that 48% of non-obstructive azoospermia had maturation arrest (18). Haddad et al., reported that HYPO with the frequency of 55.8% was the most common histological pattern in the cases with non-obstructive infertility (19). Also, the study by Thomas et al., in Nigeria reported the prevalence of HYPO pattern among 19% of the cases (20). Although the most common histological pattern was so variable in different studies, these studies were mostly conducted on the cases with non-obstructive azoospermia. The pregnancy rate was higher among the wives of males with obstructive infertility than the ones with non-obstructive infertility. Varnaeve et al., showed that conception was much lower in the cases with non-obstructive infertility than the ones with obstructive infertility (48.5% vs. 59.7%). Also, embryos replacement and clinical pregnancy were considerably lower in the cases with non-obstructive infertility than those with obstructive infertility (8.6% vs. 12.5% and 15.4% vs. 24%, respectively). It seems that the quantity and quality of spermatozoids in the cases with non-obstructive azoospermia was low. The rate of pregnancy in patients with non-obstructive infertility caused by varicocele was 5%; however, de novo testis abnormality leads to low success rate. The 23-year investigation on the histological patterns of testicular biopsy in Northern Iran was one of the main advantages of the present study. Some of limitations of the current study were the lack of evaluation of the duration of infertility, primary infertility, and considering FSH and LH status.

## Conclusion

Based on the current study results, the most common histological pattern of testicular biopsy during 23 years was SCO. Pure patterns were more than mixed patterns and the mean age of the cases was lower in mixed patterns. Also, pure patterns were the most common findings in the obstructive cases.
